# Order-of-Mutation Effects on Cancer Progression: Models for Myeloproliferative Neoplasm

**DOI:** 10.1007/s11538-024-01257-5

**Published:** 2024-02-16

**Authors:** Yue Wang, Blerta Shtylla, Tom Chou

**Affiliations:** 1 Department of Computational Medicine, UCLA, Los Angeles, CA 90095 USA; 2 Department of Statistics, Irving Institute for Cancer Dynamics, Columbia University, New York, NY 10027 USA; 3 Mathematics Department, Pomona College, Claremont, CA 91711 USA; 4 Pharmacometrics and Systems Pharmacology, Pfizer Research and Development, San Diego, CA 92121 USA; 5 Department of Mathematics, UCLA, Los Angeles, CA 90095 USA

**Keywords:** Cancer, Bistability, Mutation order, Gene expression, Moran process

## Abstract

In some patients with myeloproliferative neoplasms (MPN), two genetic mutations are often found: JAK2 V617F and one in the TET2 gene. Whether one mutation is present influences how the other subsequent mutation will affect the regulation of gene expression. In other words, when a patient carries both mutations, the *order* of when they first arose has been shown to influence disease progression and prognosis. We propose a nonlinear ordinary differential equation, the Moran process, and Markov chain models to explain the non-additive and non-commutative mutation effects on recent clinical observations of gene expression patterns, proportions of cells with different mutations, and ages at diagnosis of MPN. Combined, these observations are used to shape our modeling framework. Our key proposal is that bistability in gene expression provides a natural explanation for many observed order-of-mutation effects. We also propose potential experimental measurements that can be used to confirm or refute predictions of our models.

## Introduction

Although the timing and probability of multiple random mutations in the context of cancer have been well-studied within the classic “two-hit” (Knudsen 1971) and “multi-hit” (Armitage and Doll 2004; Bozic et al. 2010; Chou and Wang 2015) stochastic models, these constructs do not distinguish the order in which different mutations are acquired within an individual. While a multi-hit model has recently been extended to enumerate mutations according to order of appearance in colorectal cancer (KRAS, APC and TP53) (Li et al. 2023) and to compute probabilities of specific mutation trajectories, it does not address gene interactions or the mechanisms behind order-of-mutation effects. However, recent data derived from patients with myeloproliferative neoplasm (MPN), a cancer of the bone marrow (Ortmann et al. 2015), show unexpected and complex effects of mutation order on gene expression, cell populations, and patient prognosis. These observations hint at richer downstream mechanisms. We shall address these puzzles with models and analyses that provide insight into the mechanisms surrounding mutation trajectories in MPN.

Two common mutations arise in MPN: JAK2 V617F (henceforth abbreviated as JAK2) and TET2. These mutations are known to have different effects on cell behavior (Levine and Gilliland 2007; Delhommeau et al. 2009; Klampfl et al. 2013; Nangalia et al. 2013). JAK2, the Janus kinase 2, mediates cytokine signaling to control blood cell proliferation, while the “downstream” TET2 protein catalyzes oxidation of 5-methylcytosine, thereby epigenetically influencing expression of other genes. It has been shown *in vitro* that JAK2 mutations confer a competitive growth advantage in some myeloid cells (Baik et al. 2021). Similarly, TET2 mutations affect expression of target genes, leading to hematologic malignancies (Chiba 2017). This genetic disregulation is thought to indirectly increase cancer cell proliferation through a change in phenotyptic switching rates (Morinishi et al. 2020). Overall, once the JAK2 or TET2 mutation appears in certain myeloid cells, these cells effectively have higher proliferation rates than myeloid cells without such mutations. This growth advantage is modest and might take years to manifest itself as an increased proportion of cells carrying these mutations. Thus, it is common to find cells in a patient with different numbers of mutation types. Moreover, in certain patients with *both* JAK2 and TET2 mutations, it is possible to infer which mutation appears first.

Ortmann et al. (2015) reported that different mutational patterns (including the order of mutations) in hematopoietic cells and progenitor cells are related to differences in gene expression patterns, clonal evolution, and even macroscopic properties. Specifically, a mutation can differentially regulate gene expression by different amounts depending on whether another type of mutation preceded it. Therefore, the change in gene expression level when one mutation appears cannot simply be added. We call such phenomena “**non-additivity**.” Additionally, patients in which the JAK2 mutation appears before the TET2 mutation have different gene expression levels, percentages of cells with only one mutation, and ages at diagnosis than patients in which the TET2 mutation appears before the JAK2 mutation. This observation implies that the order of the first appearance of these two mutations matters. We describe such phenomena as “**non-commutative**.”

In this paper, we develop a conceptual framework based on simple dynamical and statistical models that can explain the clinically observed features associated with MPN. By hypothesizing that MPN qualitatively follows our proposed dynamics, our analysis can provide predictions that can be tested. Moreover, our nonlinear physical models are sufficiently simple and general enough that they can be used to model other cancers (Li et al. 2023) and other processes such as cellular adaptation (Brooks et al. 2011).

Before delving into our modeling and analysis, we first summarize in the next section the key clinical observations and measurements (modeling constraints) reported by Ortmann et al. (Ortmann et al. 2015). In Sect. 3, we build nonlinear ordinary differential equation (ODE) models that will serve as building blocks to explain the observed non-additivity and non-commutivity of gene expression levels under different mutation states. Variants of our model are then adapted to specific observations in the subsequent subsections. The known experimental features are consistent with our models. To explain the observations regarding clonal evolution and ages at diagnosis, we propose in Sect. 4 three different mechanisms and study them using a generalized Moran process. We conclude with some discussion in Sect. 5, including a comparison with previously proposed models, which are further detailed in Appendix A. Appendix B provides an alternative, but related Markov chain model to explain non-commutative effects of mutations on gene expression.

## Clinical Observations on the Effects Mutation Order

For patients exhibiting cells with *both* JAK2 and TET2 mutations, one might ask: Which mutation occurred first in the patient? If we find cells with only JAK2 mutations, cells with both JAK2 and TET2 mutations, but no cells with only the TET2 mutation, then the JAK2 mutation must have appeared in the patient before the TET2 mutation. Such patients are classified as JAK2-first. Patients in which we find doubly mutated cells and TET2-only cells but not JAK2-only cells are classified as TET2-first. If a patient carries JAK2-only cells, TET2-only cells, and JAK2-TET2 cells, then both JAK2 and TET2 mutations occurred independently in wild-type cells and more information, such as other associated mutations or tagging that resolves subpopulations, is needed to infer their temporal order of appearance. Such patients were not considered by Ortmann et al. (2015). For more complex samples that contain cells with multiple types of mutations, one can use different algorithms to determine the probabilities of different orders of mutations from sequencing data (De Bie et al. 2018; Pellegrina and Vandin 2022; Ramazzotti et al. 2019; Khakabimamaghani et al. 2019; Gao et al. 2022). However, patients with ambiguous cell populations (JAK2-only cells, TET2-only cells, and JAK2-TET2 cells) were not considered by Ortmann et al. (2015).

Besides inferring the order of mutations, Ortmann et al. (2015) also measured bulk gene expression levels from MPN-patient-derived populations of cells containing different sets of mutations. Their observations are summarized in Table 1 in which $x^*$ denotes the steady state expression level of gene X in a cell and the subscripts define mutation status of the cell. A majority of the data are the measured cellular expression values $x^{*}$ of a number of genes under different mutation states. We enumerate the main observations below:

**Table 1:** Definition of stationary gene expression levels $x^*$ for cells with different mutation patterns

	w/o JAK2 mutation	with JAK2 mutation
w/o TET2 mutation	$x^*_\text {O}$	$x^*_\text {J}$
with TET2 mutation	$x^*_\text {T}$	$x^*_\text {JT}$ (JAK2-first) $x^*_\text {TJ}$ (TET2-first)

**(1)** Some genes are up-regulated (or down-regulated) by a JAK2 mutation only if the TET2 mutation is not present. If the TET2 mutation is also present, the expression of these genes is not affected. Quantitatively, these observations mean $x^*_\text {T}=x^*_\text {TJ}$, $x^*_\text {O}> x^*_\text {J}$ or $x^*_\text {O}< x^*_\text {J}$ for the corresponding genes.

**(2)** Other genes are up-regulated (or down-regulated) by JAK2 mutations only if TET2 mutations are also present, but they are not affected if the TET2 mutation is *not* present. For these cases, $x^*_\text {O}=x^*_\text {J}$, $x^*_\text {J}>x^*_\text {TJ}$ or $x^*_\text {J}<x^*_\text {TJ}$.

**(3)** Ten genes (AURKB, FHOD1, HTRA2, IDH2, MCM2, MCM4, MCM5, TK1, UQCRC1, WDR34) are up-regulated in cells with JAK2 mutations if TET2 mutations are not present, but they are down-regulated by JAK2 mutations if TET2 mutations *are* present. This scenario corresponds to $x^*_\text {O}< x^*_\text {J}$, $x^*_\text {T}> x^*_\text {TJ}$.

**(4)** Different orders of appearances of JAK2 and TET2 mutations seem to have different effects on other genes so that $x^*_\text {JT}\ne x^*_\text {TJ}$. These conclusions are inferred from other indirect evidence (e.g., JAK2-first cells are more sensitive to ruxolitinib than TET2-first cells (Ortmann et al. 2015)).

Observations **(1–3)** can be regarded as **non-additivity** since the effect of JAK2 mutation differs with or without TET2 mutation. In other words, $x^*_\text {J}- x^*_\text {O}\ne x^*_\text {TJ}- x^*_\text {T}$. Observation **(4)** represents **non-commutativity** since exchanging the order of acquiring different mutations can lead to different expression levels or cell states (Levine et al. 2019). Mathematically, $x^*_\text {O}+(x^*_\text {J}-x^*_\text {O})+(x^*_\text {JT}-x^*_\text {J})=x^*_\text {JT}\ne x^*_\text {TJ}=x^*_\text {O}+(x^*_\text {T}-x^*_\text {O})+(x^*_\text {TJ}-x^*_\text {T})$. In fact, if the gene expression levels are additive with respect to multiple mutations, namely $x^*_\text {J}- x^*_\text {O}= x^*_\text {TJ}-x^*_\text {T}$ and $x^*_\text {T}-x^*_\text {O}= x^*_\text {JT}-x^*_\text {J}$, then these multiple mutations are also commutative with respect to order: $x^*_\text {JT}= x^*_\text {J}+x^*_\text {T}-x^*_\text {O}=x^*_\text {TJ}$. Therefore, non-commutativity is a special case of non-additivity. At the cell and patient level, Ortmann et al. (2015) also report two observations specifically related to non-commutativity.

**(5)** In TET2-first patients, the percentage of cells with just one mutation (TET2) is significantly higher than the percentage of JAK2-only cells in JAK2-first patients.

**(6)** At diagnosis, JAK2-first patients are significantly younger than TET2-first patients.

Ortmann et al. (2015) also report other observations such as differences in MPN classification and risk of thrombosis between JAK2-first and TET2-first patients. These are implicitly covered by observations **(1–6)**, particularly **(4)**, and we do not explicitly discuss them here.

## Models for Non-additivity and Non-commutativity in Gene Expression

In this section, we propose the theoretical building blocks that provide mechanistic explanations for observations **(1, 2, 3, 4)**, emphasizing the non-additive and non-commutative properties of the mutations on gene expression.

### Mathematical Background

First, consider simple ordinary differential equation (ODE) models for gene expression and regulation. For gene X with expression level *x*(*t*), the simplest model $\textrm{d}x/\textrm{d}t=\lambda -\gamma x$ considers only synthesis and degradation with constant rates $\lambda , \gamma$, and a stationary state $x^*=\lambda /\gamma$. If other genes (mutations) regulate the expression of X, we can allow the synthesis rate $\lambda$ to depend on other factors, which may include the activation state of genes Y and Z. For example, we might write a deterministic model for the expression level *x*(*t*) as

1 $$\begin{aligned} \frac{\textrm{d}x(t)}{\textrm{d}t}= \lambda _{0} + \lambda _\textrm{Y}\mathbb {1}_{\textrm{Y}} + \lambda _\textrm{Z}\mathbb {1}_{\textrm{Z}}-\gamma x. \end{aligned}$$

Here, we have modeled the synthesis rate $\lambda = \lambda _{0} + \lambda _\textrm{Y}\mathbb {1}_{\textrm{Y}}+ \lambda _\textrm{Z}\mathbb {1}_{\textrm{Z}}$ as a Boolean control operator by using a discrete indicator function where $\mathbb {1}_{\textrm{Y}}=1$ if Y (gene activity or product) is present, $\mathbb {1}_{\textrm{Y}}=0$ otherwise, and $\lambda _\textrm{Y}$ is a constant regulation amplitude of gene Y on the expression of gene X. A similar term with amplitude $\lambda _\textrm{Z}$ arises for mutation Z. After $\mathbb {1}_{\textrm{Y}}$ or $\mathbb {1}_{\textrm{Z}}$ changes (e.g., one gene mutates), the expression level of X will eventually return to a new equilibrium. Therefore, in this section, we consider only the stationary state $x^*$.

The linear (in *x*) ODE in Eq. 1 cannot explain observations **(1–4)** since the regulation effects of different genes (mutations) would be additive and commutative. Regardless of the status of other genes and mutations, the presence or absence of either mutation always has the same effect. In order to generate nonadditive or noncommutative effects, one needs to at minimum incorporate a nonlinear or “gene regulatory” term. Consider the general form

2 $$\begin{aligned} \frac{\textrm{d}x(t)}{\textrm{d}t}= \lambda + f(x)-x, \end{aligned}$$

where for simplicity we have normalized time so that the intrinsic degradation rate $\gamma \equiv 1$ and $\lambda$ is the dimensionless synthesis rate that may still depend on the presence of mutations of other genes (thus being “tuned” by mutations). The nonlinear term *f*(*x*) represents the autoregulation of *X* (Wang and He 2023). While many possible forms for *f*(*x*) may be inferred from measurements or otherwise approximated or modeled, particularly common and useful are Hill functions of the form $x^{n}/(x^{n}+C^{n})$. Although mathematical models have yet to be developed specifically for JAK2 and TET2 expression dynamics, many related models of gene expression that use very similar nonlinear self-regulation terms of the Hill type have been developed (Mackey et al. 2016; Dresch et al. 2013). Specifically, mathematical models that reflect measured expression dynamics associated with the JAK2-STAT pathway have included nonlinear terms of the Hill form (Lee et al. 2021). For TET2, a model that includes its action on DNA methylation gives rise to a Hill-type nonlinear term for TET2 expression (Chen et al. 2021). Such a saturating *f*(*x*), along with an appropriate decay term $-x$, leads to a form of $\lambda +f(x)-x$ that may exhibit up to three zeros, depending on the constant value of $\lambda$. Thus, following the spirit of these related models, we will choose an $f(x)-x$ that follows a similar nonmonotonic shape (decreasing, increasing, then decreasing with respect to *x*):

3 $$\begin{aligned} f(x) =-(x-2)^3+2(x-2). \end{aligned}$$

**Fig. 1:**
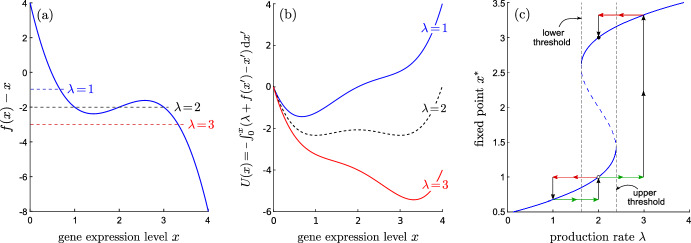
**a** A dimensionless autoregulation interaction and decay term $f(x)-x$ (blue curve). Zeros of the right-hand side of Eq. 2 for different values of the production control parameter $\lambda$ (0,2,4) are indicated. **b** By writing the right-hand side of Eq. 2 as a gradient $-\partial _{x} U(x)$, we can define the associated potential *U*(*x*). As $\lambda$ changes, the potential develops minima at different values of *x*. **c** The potential-minimizing equilibrium values of expression levels of gene X, $x^{*}$, are plotted against $\lambda$ showing two stable branches: a low-value one and a high-value one (solid blue curves). The dashed blue curve traces out the unstable branch. Gene expression that is non-additive and non-commutative in mutations that change $\lambda$ is a natural consequence of the dynamics proposed in this model. Suppose the system starts in the low-$x^{*}$ branch (open circle) and the mutation order is such that $\lambda =2 \rightarrow \lambda = 3 \rightarrow \lambda = 2$ and $\lambda =2 \rightarrow \lambda = 1 \rightarrow \lambda = 2$, with both scenarios ending at $\lambda = 2$. If $\lambda$ is first increased (shown by the green arrows) and then decreased (red arrows), the system arrives at the high-$x^{*}$ branch (black dot). However, if $\lambda$ is first decreased then increased, the system remains in the low-$x^{*}$ branch (Color figure online)

We choose this simple form because $f(x)-x$ has the same qualitative features that can lead to three explicit roots (and bistability), allowing for a simpler, concrete analysis. Figure 1a shows $f(x)-x$ as well as its values that would balance certain values of $\lambda$ to make $\textrm{d}x(t)/\textrm{d}t = 0$. The corresponding potentials *U*(*x*) are shown in Fig. 1b for $\lambda = 0,2,4$. The fixed points (stationary states) of Eq. 2 are plotted in Fig. 1c as a function of $\lambda$ and show the high- and low-expression level branches.

For this nondimensionalized model, when $\lambda < 1.6$, there is one stable, low-value fixed point near $x^{*}\lesssim 0.8$. If $\lambda >2.4$, there is one stable fixed point near $x^{*} \gtrsim 3.2$ that defines the stable high-value branch. At intermediate values $1.6< \lambda <2.4$, both values of $x^*$ (high and low) are locally stable and are connected by an unstable middle branch of fixed points (dashed curve).

When we start from $\lambda <1.6$, the system resides only on the low expression level branch. If $\lambda$ is then increased to $1.6<\lambda <2.4$, even though there are two stable branches, the system remains in the low-$x^{*}$ branch. When we further increase $\lambda$ until $\lambda >2.4$, the stable low-$x^{*}$ branch and the unstable middle branch collide and disappear (saddle-node bifurcation) and the system jumps to the stable high-$x^{*}$ branch. If we start with $\lambda >2.4$, the system is in the high-level branch. Decreasing $\lambda$ to $1.6<\lambda <2.4$, the system will stay in the stable high-level branch until $\lambda <1.6$, at which point the stable high-level branch and the unstable intermediate-value branch collide and disappear, and the system jumps to the low-$x^{*}$ branch. In this model, when we change the parameter $\lambda$ along different trajectories, the final stationary state can differ even though all trajectories $\lambda (t)$ arrive at the same final values within $1.6<\lambda <2.4$. For example, if the value of $\lambda$ is evolved according to $\lambda =2\rightarrow \lambda =1\rightarrow \lambda =2$, the final state is $x^*=1$, but if $\lambda$ follows the trajectory $\lambda =2\rightarrow \lambda =3\rightarrow \lambda =2$, the final state is the high-value one at $x^*=3$.

Now consider a model in which the source of *X* is controlled by genes Y and Z through $\lambda = \lambda _{0} + \lambda _\textrm{Y} \mathbb {1}_\textrm{Y} + \lambda _\textrm{Z}\mathbb {1}_\textrm{Z}$. Genes Y and Z can qualitatively affect the stationary state values of expression of X, $x^{*}$, if including their presence (or absence) induces $\lambda$ to cross the thresholds at 1.6 and 2.4. This model structure means that different orders of mutations (changes in Y and Z) can give rise to different stationary states and lead to non-additive and non-commutative effects on X.

An additional important ingredient must be noted. Models such as Eqs. 2 and 3 are typically applied to gene expression within a single cell. Even though mutations can also change the regulation function *f*(*x*), we have focussed only on how they change $\lambda$. The bistability of this system implies that mutations or different combinations of mutations can drive the system from one branch to another. However, under normal conditions, individual cells do not acquire mutations which typically arise during DNA replication as part of cell proliferation. At birth, one daughter cell can acquire a new mutation with some probability. Therefore, the effects of mutations on $\lambda$ can be cumulative only if the values of $\lambda$ *are transmitted across lineages*. In other words, the epigenetic state of a mother cell that supports a certain value of $\lambda$ must be inherited by the daughter cells, which may then acquire a new mutation that further changes $\lambda$. This epigenetic inheritance operates across the eukaryotic cell cycle (Probst et al. 2009) and is mechanistically motivated by e.g., factors that copy methylation patterns across complementary DNA strands during replication (Vandiver et al. 2016). Given this reasonable assumption, we now use the mathematical structure given in Eq. 2 to explore the different behaviors of various measured genes and to explain observations **(1, 2, 3, 4)**. Note that we just need Eq. 2 to be nonlinear (to generate non-additivity) and to exhibit bistability (to induce non-commutativity).

### TET2-Gated Regulation in a JAK2 Mutation Background [Observations **(1, 2)**]

**Fig. 2:**
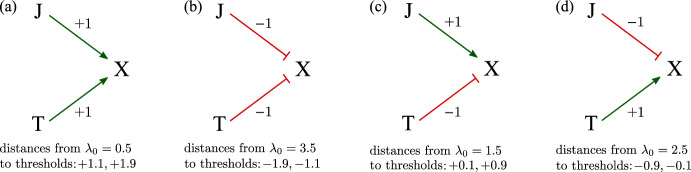
**a** A schematic of the model $\lambda =0.5+\mathbb {1}_\textrm{J}+\mathbb {1}_\textrm{T}$ that yields $x^*_\text {O}=x^*_\text {J}$ and $x^*_\text {T}<x^*_\text {TJ}$. “J $\longrightarrow$ X” indicates that the presence of a J mutation up-regulates expression of X. In this particular model, we have $x^*_\text {O}\approx 0.6$, $x^*_\text {J}\approx 0.8$, $x^*_\text {T}\approx 0.8$, and $x^*_\text {TJ}\approx 3.2$. **b** Schematic of the model $\lambda =3.5-\mathbb {1}_\textrm{J}-\mathbb {1}_\textrm{T}$ which yields $x^*_\text {O}=x^*_\text {J}$ but $x^*_\text {T}>x^*_\text {TJ}$. “J,T  X” indicates that JAK2 and TET2 mutations both down-regulate X. Here, we have $x^*_\text {O}\approx 3.4$, $x^*_\text {J}\approx 3.2$, $x^*_\text {T}\approx 3.2$, $x^*_\text {TJ}\approx 0.8$. **c** The production rate model $\lambda =1.5+\mathbb {1}_\textrm{J}-\mathbb {1}_\textrm{T}$ captures $x^*_\text {O}<x^*_\text {J}$, $x^*_\text {T}=x^*_\text {TJ}$. Here, we have $x^*_\text {O}\approx 0.8$, $x^*_\text {J}\approx 3.2$, $x^*_\text {T}\approx 0.6$, $x^*_\text {TJ}\approx 0.8$. **d** $\lambda =2.5-\mathbb {1}_\textrm{J}+\mathbb {1}_\textrm{T}$ explains $x^*_\text {O}>x^*_\text {J}$ but maintains $x^*_\text {T}=x^*_\text {TJ}$. As before, the symbols “$\longrightarrow$” and “” represent up-regulation and down-regulation, respectively. This scenario yields $x^*_\text {O}\approx 3.2$, $x^*_\text {J}\approx 0.8$, $x^*_\text {T}\approx 3.4$, $x^*_\text {TJ}\approx 3.2$. The distances of the lower and upper thresholds to the value of $\lambda _{0}$ are indicated for all cases

We consider different variants of Eq. 2 to explain why some genes follow $x^*_\text {O}\ne x^*_\text {J}$, but others obey $x^*_\text {T}= x^*_\text {TJ}$ (and vice versa). In the following, “J” will indicate the JAK2 mutation while “T” will denote the TET2 mutation. In this application, Y and Z are identified as target genes regulated by J and T. Thus, we can simplify the expression rate $\lambda$ in Eq. 2 to, e.g., $\lambda =0.5+\mathbb {1}_\textrm{J}+\mathbb {1}_\textrm{T}$. With no mutation, $\lambda =\lambda _{0}=0.5$, and the system is in the low-expression state $x^*_\text {O}\approx 0.6$. Consider a scenario in which $\mathbb {1}_{\textrm{T}}=0$, $\mathbb {1}_{\textrm{J}}=1$, i.e., the JAK2 mutation is present but not the TET2 mutation (or vice versa). Then, $\lambda =1.5$ and the system is in the low-$x^{*}$ state $x^*_\text {J}\approx 0.8$ (also, $x^*_\text {T}\approx 0.8$). If both JAK2 and TET2 mutations are present, then $\mathbb {1}_{\textrm{T}}=\mathbb {1}_{\textrm{J}}=1$, $\lambda =2.5$, and the system is in the high-$x^{*}$ state $x^*_\text {TJ}\approx 3.2$. We have $x^*_\text {O}\approx x^*_\text {J}$ but $x^*_\text {T}< x^*_\text {TJ}$. Therefore, in this case, JAK2 mutation up-regulates X only if the TET2 mutation is present. See Fig. 2a for an illustration of this scenario.

Now, assume the regulated production rate takes the form $\lambda =3.5-\mathbb {1}_\textrm{J}-\mathbb {1}_\textrm{T}$. If $\mathbb {1}_{\textrm{T}}=0$ (no TET2 mutation), then $\lambda =3.5$ and a JAK2 mutation itself does not affect X expression much ($x^*_\text {J}\approx 3.2$, $x^*_\text {O}\approx 3.4$). If $\mathbb {1}_{\textrm{T}}=1$, then a JAK2 mutation (changing $\mathbb {1}_{\textrm{J}}$ from 0 to 1) will alter the *x*-production rate to $\lambda =1.5$ which is sufficient to decrease the steady state expression level from $x^*_\text {T}\approx 3.2$ to $x^*_\text {TJ}\approx 0.8$. While $x^*_\text {O}\approx x^*_\text {J}$, $x^*_\text {T}> x^*_\text {TJ}$. Thus, the JAK2 mutation down-regulates expression of X only if the TET2 mutation is present. See Fig. 2b for a schematic of this scenario.

Now, if $\lambda =1.5+\mathbb {1}_\textrm{J}-\mathbb {1}_\textrm{T}$, then if the T is absent (no TET2 mutation), the presence of J (a JAK2 mutation) up-regulates X since $x^*_\text {O}\approx 0.8$, $x^*_\text {J}\approx 3.2$. In the presence of T, J does not affect X expression much since $x^*_\text {T}\approx 0.6$, $x^*_\text {TJ}\approx 0.8$. This regulation model is depicted in Fig. 2c.

Finally, consider a gene expression rate governed by $\lambda =2.5-\mathbb {1}_\textrm{J}+\mathbb {1}_\textrm{T}$, as shown in Fig. 2d. If T is not present, then J down-regulates X since $x^*_\text {O}\approx 3.2$, $x^*_\text {J}\approx 0.8$. In the presence of T, J does not affect X expression much since $x^*_\text {T}\approx 3.4$, $x^*_\text {TJ}\approx 3.2$.

### TET2 Mutation Inverts Expression Under a JAK2 Mutationbackground [Observation (3)]

**Fig. 3:**
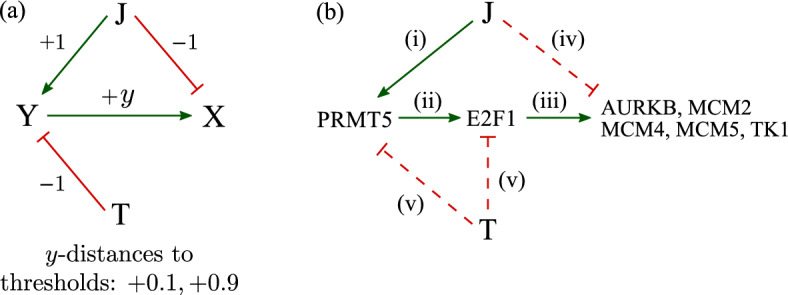
**a** Schematic of a model that explains $x^*_\text {O}<x^*_\text {J}$ but $x^*_\text {T}>x^*_\text {TJ}$. In this model, the steady-state expression levels of Y are $y^*_\text {O}\approx 0.8$, $y^*_\text {J}\approx 3.2$, $y^*_\text {T}\approx 0.6$, $y^*_\text {TJ}\approx 0.8$. The basal value of $x=1$, while the different stationary expression levels of X are $x^*_\text {O}\approx 1.8$, $x^*_\text {J}\approx 3.2$, $x^*_\text {T}\approx 1.6$, $x^*_\text {TJ}\approx 0.8$. **b** The gene regulatory network that explains $x^*_\text {O}<x^*_\text {J}$ but $x^*_\text {T}>x^*_\text {TJ}$ for a number of genes. Solid line indicates a verified regulation while the dashed line denotes a hypothesized regulatory interaction

To explain observation **(3)** that $\text {O}\rightarrow \text {J}$ and $\text {T}\rightarrow \text {TJ}$ have opposite effects, we need a more complicated variant of Eq. 2. Consider a gene Y whose expression level *y* is described by

4 $$\begin{aligned} \frac{\textrm{d}y}{\textrm{d}t}=\lambda +f(y)-y, \end{aligned}$$

in which $\lambda =1.5+\mathbb {1}_\textrm{J}-\mathbb {1}_\textrm{T}$ and $f(y)=-(y-2)^3+2(y-2)$. This setup gives rise to $y^*_\text {O}\approx 0.8$, $y^*_\text {T}\approx 0.6$, $y^*_\text {TJ}\approx 0.8$, and $y^*_\text {J}\approx 3.2$. Now, consider a gene X whose expression level follows the linear dynamics

5 $$\begin{aligned} \frac{\textrm{d}x}{\textrm{d}t}=1-\mathbb {1}_\textrm{J}+y-x, \end{aligned}$$

where X has a basal synthesis and decay rate of 1. A JAK2 mutation can directly down-regulate X expression with strength 1, while expression of Y can up-regulate that of X with strength proportional to its expression level *y*. Figure 3a shows the key regulation processes in this model. Without JAK2 and TET2 mutations, $\lambda =1.5$, which is under the lower threshold of $\lambda =1.6$. In this case, Y is in its low-expression state $y^*_\text {O}\approx 0.8$ and X is only weakly affected by Y, with a stationary expression level $x^*_\text {O}\approx 1.8$. With J but not T, $\lambda =2.5$, which is above the upper threshold 2.4. In this case, Y is in its high-value state $y^*_\text {J}\approx 3.2$. Now, X expression is affected by both J and Y (strongly), taking on the value $x^{*}_{\text {J}} \approx 3.2$. With T but not J, $\lambda =0.5$, below the lower threshold of 1.6. In this case, Y is in its low-value state $y^*_\text {T}\approx 0.6$ and X expression, $x^*_\text {T}\approx 1.6$, is only weakly affected by Y expression.

In the presence of both JAK2 and TET2 mutations, $\lambda =1.5$, under the lower threshold of 1.6. In this case, Y is in its low-value state $y^*_\text {TJ}\approx 0.8$ and X is affected weakly by Y expression and by the JAK2 mutation, with $x^*_\text {TJ}\approx 0.8$. Therefore, without a TET2 mutation, a JAK2 mutation up-regulates X expression (from $x^*_\text {O}\approx 1.8$ to $x^*_\text {J}\approx 3.2$); with the TET2 mutation, a JAK2 mutation down-regulates X expression from $x^*_\text {T}\approx 1.6$ to $x^*_\text {TJ}\approx 0.8$.

To explain observation **(3)**, the proposed model (Eqs. 4 and 5) introduces an extra gene Y in order to explain $x^*_\text {O}<x^*_\text {J}$ and $x^*_\text {T}>x^*_\text {TJ}$. Potential candidates for Y are revealed by the structure of our proposed model, allowing it to describe gene expression levels measured to date as long as five interactions/regulatory dependences are satisfied:

(i) In MPN, the expression of PRMT5 is higher in cells with the JAK2 V617F mutation (Pastore et al. 2020).
(ii) PRMT5 inhibition reduces the expression of E2F1. Thus, PRMT5 up-regulates E2F1 (Pastore et al. 2020).
(iii) The expression of E2F1 induces all genes of the endogenous MCM family (Ohtani et al. 1999). E2F1 is a transcriptional activator of AURKB (Yu et al. 2017) that can up-regulate AURKB and MCM5 expression (Reyes et al. 2018). Overexpressing E2F1 alone results in the up-regulation of MCM5 and TK1 (Koushyar et al. 2017). In sum, E2F1 up-regulates AURKB, MCM2, MCM4, MCM5, and TK1.
(iv) A JAK2 mutation can weakly but directly down-regulate AURKB, MCM2, MCM4, MCM5, and TK1.
(v) A mutated TET2 gene can down-regulate E2F1 directly, or indirectly through PRMT5. This down-regulation cancels out the up-regulation JAK2 $\rightarrow$ PRMT5 $\rightarrow$ E2F1. This means E2F1 (and possibly PRMT5) expression satisfies $y^*_\text {J}> y^*_\text {JT}$ and $y^*_\text {O}>y^*_\text {T}$.

As referenced, effects (i)–(iii) have been directly experimentally verified, while mechanisms (iv) and (v) are assumptions that provide consistency between our model and observations; thus, (iv) and (v) can also be considered predictions of our simple modeling approach.

Ortmann et al. (2015) reported ten genes that follow $x^*_\text {O}<x^*_\text {J}$ but also $x^*_\text {T}> x^*_\text {TJ}$: AURKB, FHOD1, HTRA2, IDH2, MCM2, MCM4, MCM5, TK1, UQCRC1, and WDR34. Our model can explain five of them (AURKB, MCM2, MCM4, MCM5, TK1) with the same pathway JAK2 $\rightarrow$ PRMT5 $\rightarrow$ E2F1 $\rightarrow$ AURKB/MCM2/MCM4/MCM5/TK1, while the role of Y can be played by E2F1 and/or PRMT5.

Figure 3b shows a simple gene regulatory network that is consistent with the observations. In patients without a TET2 mutation, the JAK2 mutation can up-regulate PRMT5 and E2F1, which in turn up-regulate AURKB, MCM2, MCM4, MCM5, and TK1; this strong indirect up-regulation of JAK2 $\rightarrow$ PRMT5 $\rightarrow$ E2F1 $\rightarrow$ AURKB/MCM2/MCM4/MCM5/TK1 can cover the weak direct down-regulation JAK2 $\dashv$ AURKB/MCM2/MCM4/MCM5/TK1, and the overall effect is $x^*_\text {O}<x^*_\text {J}$. In the presence of the TET2 mutation, the up-regulation JAK2 $\rightarrow$ PRMT5 $\rightarrow$ E2F1 is covered by the down-regulation TET2 $\dashv$ PRMT5/E2F1; therefore, PRMT5 and E2F1 are locked to low levels so that the only effective regulation of JAK2 is the down-regulation JAK2 $\dashv$ AURKB/MCM2/MCM4/MCM5/TK1.

Interpreting the observation using our simple model suggests two properties: the JAK2 mutation weakly but directly down-regulates certain genes (AURKB, MCM2, MCM4, MCM5, and TK1); E2F1 (and possibly PRMT5) expression satisfies $y^*_\text {J}> y^*_\text {JT}$ and $y^*_\text {O}>y^*_\text {T}$. In principle, the first hypothesis can be experimentally verified by introducing a JAK2 mutation after knockdown or knockout of PRMT5 or E2F1 and observing decreased AURKB, MCM2, MCM4, MCM5, and TK1 expression. The prediction that E2F1 expression satisfies $y^*_\text {J}> y^*_\text {JT}$ and $y^*_\text {O}>y^*_\text {T}$ can be tested by comparing its expression level in cells with and without the TET2 mutation. Lower levels of E2F1 (and possibly PRMT5) in cells with a TET2 mutation would be consistent with our the network shown in Fig. 3b.

The pathway JAK2 $\rightarrow$ PRMT5 $\rightarrow$ E2F1 $\rightarrow \cdots$ is but one possibility. There is also evidence for the role of p53 in observation **(3)**. JAK2 V617F negatively regulates p53 stabilization (Nakatake et al. 2012), while p53 can regulate AURKB and MCM5 (Reyes et al. 2018). The complete gene regulatory network should be determined using certain inference methods based on gene expression data (Wang and Wang 2022; Bocci et al. 2022).

### Different Orders of Mutation Yield Different Expression Levels [Observation (4)]

**Fig. 4:**
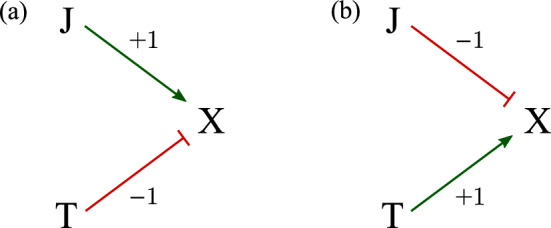
**a** A schematic of the model $\lambda =2+ \mathbb {1}_{\textrm{J}}- \mathbb {1}_{\textrm{T}}$ in Eq. 2 which explains $x^*_\text {TJ}<x^*_\text {JT}$. If the effects of JAK2 and TET2 mutations towards the input $\lambda$ are together greater than 0.4 (i.e., with JAK2 but not TET2), the system is forced to be on the high-$x^{*}$ branch; if the contribution to $\lambda$ input JAK2 and TET2 is smaller than $-0.4$ (i.e., with TET2 but not JAK2), the system ends up on the low-value branch. **b**) The model $\lambda =2- \mathbb {1}_{\textrm{J}}+ \mathbb {1}_{\textrm{T}}$ can yield $x^*_\text {JT}<x^*_\text {TJ}$. If the contribution from JAK2 and TET2 mutations to $\lambda$ is greater than 0.4 (i.e., with TET2 but not JAK2), the system is forced onto the high-$x^{*}$ branch; if the JAK2 and TET2 contributions to the input $\lambda$ is smaller than $-0.4$ (i.e., with JAK2 but not TET2), the system is forced onto the low-$x^{*}$ branch

To explain observation **(4)** that TJ and JT have different effects, namely $x^*_\text {TJ}\ne x^*_\text {JT}$, consider Eq. 2 with $\lambda =2+ \mathbb {1}_{\textrm{J}}- \mathbb {1}_{\textrm{T}}$. With J but not T, $\lambda =3$ and X lies in its only high-value stationary state $x^*_\text {J}\approx 3.3$; if T appears after J, then $\lambda =2$, and X remains in its high-value branch with stationary level $x^*_\text {JT}=3$. If the TET2 mutation arises without a JAK2 mutation, $\lambda =1$ and the steady-state expression of X is $x^*_\text {T}\approx 0.7$; if J appears after T, then $\lambda =2$ and X expression remains in its low-value branch with stationary value $x^*_\text {TJ}=1$. See Fig. 1 for a more detailed description. For MPN patients, if the order is JT, the final X expression is high ($x^*_\text {JT}=3$); if the order is TJ, the final X expression level is low ($x^*_\text {TJ}=1$). See Fig. 4a for an illustration of this model explaining $x^*_\text {JT}>x^*_\text {TJ}$.

To explain $x^*_\text {JT}<x^*_\text {TJ}$, consider Eq. 2 with $\lambda =2- \mathbb {1}_{\textrm{J}}+ \mathbb {1}_{\textrm{T}}$. If the mutation order is JT, the final X expression level is low ($x^*_\text {JT}=1$); if the order is TJ, the final X expression level is high ($x^*_\text {TJ}=3$). This regulation control mechanism is illustrated in Fig. 4b.

## Models for Non-commutativity in Cell Population and Diagnosis Age

It was also observed that the age at diagnosis and the populations of cancer cells depend on the order of the two mutations experienced by the patient. Clinically, the mutations are non-commutative in their effects on prognosis. In this section, we build models to explain these observations **(5, 6)**. Since these observations pertain to cell populations and timing, these models can be constructed independently of the nonlinear gene expression models developed in Sect. 3.

### Different Mechanisms for Explaining Observations (5, 6)

For observations **(5, 6)**, the cell population and age are measured at the time of diagnosis. However, it is difficult to know the time interval between acquiring the second mutation and diagnosis or to model the disease progression during this time. Therefore, we analyze observations **(5, 6)** focussing on the time at which the first double-mutation cell (cells with both JAK2 and TET2 mutations) first appears. Timing of the double mutation event is easier to model since the time interval between the first double mutation in a patient and clinical diagnosis is difficult to estimate. In order to address observations **(5, 6)** theoretically, we must redefine them in terms of the time at which both mutations first appear:

**(5’)** For TET2-first patients, at the time when the first TET2-JAK2 cell appears, the percentage of TET2-only cells is significantly higher than the percentage of JAK2-only cells at the time when the first JAK2-TET2 cell appears in JAK2-first patients.

**(6’)** For JAK2-first patients, the time at which the first JAK2-TET2 cell appears is significantly earlier than the time at which the first TET2-JAK2 cell appears for TET2-first patients.

To explain observations **(5’)** and **(6’)**, we explore three mechanistic scenarios defined by different relative rates of proliferation of the different cell types. We demonstrate via simulations that each of these three scenarios can reproduce observations **(5’)** and **(6’)**.

**(A)** Ortmann et al. (2015) propose that *cells with a JAK2 mutation have only a mild proliferation advantage while cells with a TET2 mutation (whether JAK2 is present or not) have a more significant proliferation advantage*. The model by Teimouri and Kolomeisky (2021) is relevant to this mechanism in that they assume different proliferation rates between JAK2-only mutated cells and TET2-only mutated cells, but assume equal proliferation rates for JT and TJ cells; however, they also incorporate a number of assumptions that are not satisfied in this system.

**(B)** Since different mutations generally appear with different rates (Lynch 2010), we propose a mechanism in which acquiring additional JAK2 and TET2 mutations occur at different rates. Thus, they will carry different *mutation probabilities*.

**(C)** We also explore a cooperative mutation mechanism: cells with the JAK2 mutation carry a higher mutation rate for TET2 mutation. In other words, *an existing JAK2 mutation induces an additional TET2 mutation*.

### Generalized Moran Process

**Fig. 5:**
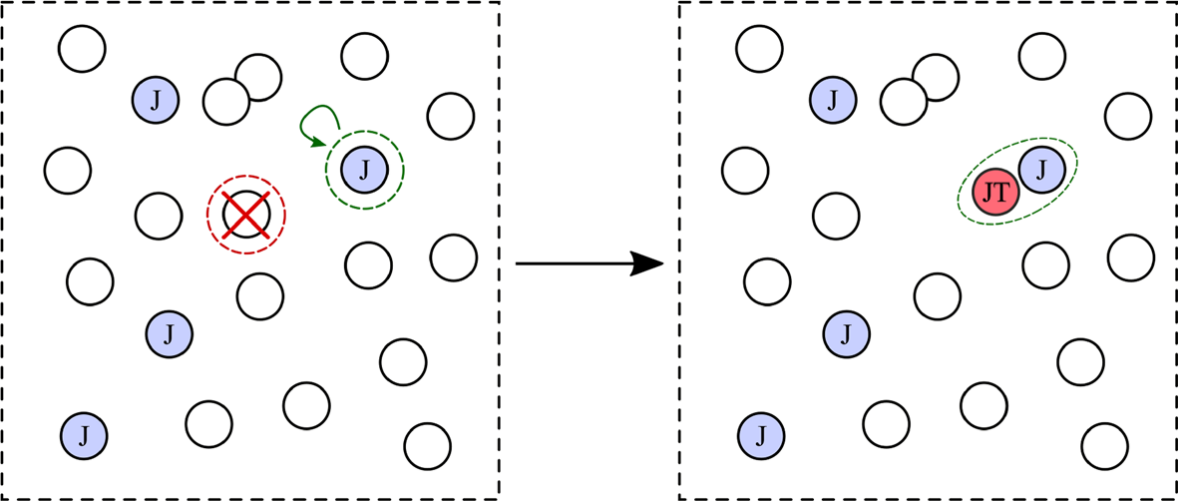
A schematic of the steps in our Moran process. In one time step, one cell (wild-type) is chosen for removal (red-dashed circle), while another (J) is chosen for replication (green-dashed circle), during which one daughter may acquire a mutation. In this example, a J cell divides into a J cell and a double-mutant JT cell, thus defining the end point of our simulation. Thus, the absorbing states of this stochastic process is defined by the presence of a single JT or TJ cell (Color figure online)

To mathematically model how mechanisms **(A)**, **(B)**, and **(C)** can give rise to observations **(5’)** and **(6’)**, we consider a generalized discrete-time Moran model (Fudenberg et al. 2004; Quan and Wang 2011), shown in Fig. 5, for cell populations that include mutations. Unlike branching processes (Jiang et al. 2017), the total number of cells is fixed in Moran processes, which is a reasonable approximation for stable hematopoietic stem cell populations. By fixing the total cell populations, we can also more easily assess relative cell populations of different mutation types. A continuous-time Moran model can also be straightforwardly constructed and analyzed. Such a Moran process for cells that can acquire two possible mutations has been discussed by Teimouri et al. (2022), but in their model, the two mutations are treated symmetrically, and mutations appear independently after cell division. A related two-mutation branching process has been used to express the first time of acquiring a double-mutation cell, but did not distinguish the order of mutation acquisition (Chou and Wang 2015).

We will assume that cells can exist in five states: non-mutant (wild-type, denoted by suffix $\text {O}$), JAK2-only (denoted by suffix $\text {J}$), TET2-only (denoted by suffix $\text {T}$), JAK2-TET2 (JAK2 appears before TET2, denoted by suffix $\text {JT}$), and TET2-JAK2 (JAK2 appears after TET2, denoted by suffix $\text {TJ}$). The state space of this process is indicated by the numbers of cells $(n_\text {O}, n_\text {J}, n_\text {T}, n_\text {JT}, n_\text {TJ})$ in each state. To describe the dynamics of this process, for cells in different states, we need the birth rate coefficients $(b_\text {O}, b_\text {J}, b_\text {T}, b_\text {JT}, b_\text {TJ})$, death rate coefficients $(d_\text {O}, d_\text {J}, d_\text {T}, d_\text {JT}, d_\text {TJ})$, and mutation probabilities $m_{\text {O}\rightarrow \text {J}}$, $m_{\text {O}\rightarrow \text {T}}$, $m_{\text {J}\rightarrow \text {JT}}$, and $m_{\text {T}\rightarrow \text {TJ}}$.

At each time step, one cell is chosen (weighted by the death coefficient of its type) for removal. For example, the probability of choosing a TET2-only cell to die is $n_\text {T}d_\text {T}/(\sum _i n_i d_i), \, i \in \{\text{ O, } \text{ J, } \text{ T, } \text{ JT, } \text{ TJ }\}$. Simultaneously, another cell is randomly (weighted by its birth coefficient) picked for division. For example, the probability of choosing a JAK2-only cell for division is $n_\text {J}b_\text {J}/(\sum _i n_i b_i) \, i \in \{\text{ O, } \text{ J, } \text{ T, } \text{ JT, } \text{ TJ }\}$. After division, one daughter cell will remain in the same state as the mother cell, while the other may acquire an extra mutation with some probability. A wild-type daughter cell can obtain a JAK2 mutation or TET2 mutation with probability $m_{\text {O}\rightarrow \text {J}}$ or $m_{\text {O}\rightarrow \text {T}}$; a JAK2-only daughter cell can obtain a TET2 mutation with probability $m_{\text {J}\rightarrow \text {JT}}$; a TET2-only daughter cell can obtain a JAK2 mutation with probability $m_{\text {T}\rightarrow \text {TJ}}$. After each elimination-replication-mutation step, the total population $\sum _i n_i =n$ remains unchanged.

We can explicitly calculate the transition probabilities of this process. At each time point, the probability that a wild-type cell is chosen for elimination is

6a $$\begin{aligned} \begin{aligned}&{\mathbb {P}}[(n_\text {O},n_\text {J},n_\text {T},n_\text {JT},n_\text {TJ})\rightarrow (n_\text {O}-1,n_\text {J},n_\text {T},n_\text {JT},n_\text {TJ})]\\&\hspace{2cm} = \frac{n_\text {O}d_\text {O}}{n_\text {O}d_\text {O}+n_\text {J}d_\text {J}+n_\text {T}d_\text {T}+n_\text {JT}d_\text {JT}+n_\text {TJ}d_\text {TJ}}. \end{aligned} \end{aligned}$$

The probability of selecting other cell types for death are similarly defined. The probability of producing an extra wild-type cell is

6b $$\begin{aligned} \begin{aligned}&{\mathbb {P}}[(n_\text {O},n_\text {J},n_\text {T},n_\text {JT},n_\text {TJ})\rightarrow (n_\text {O}+1,n_\text {J},n_\text {T},n_\text {JT},n_\text {TJ})]\\&\hspace{2cm} = \frac{n_\text {O}b_\text {O}(1-m_{\text {O}\rightarrow \text {J}}-m_{\text {O}\rightarrow \text {T}})}{n_\text {O}b_\text {O}+n_\text {J}b_\text {J}+n_\text {T}b_\text {T}+n_\text {JT}b_\text {JT}+n_\text {TJ}b_\text {TJ}}. \end{aligned} \end{aligned}$$

Similarly, the probabilities of generating additional cells of other cell types are

6c $$\begin{aligned}{} & {} \begin{aligned}&{\mathbb {P}}[(n_\text {O},n_\text {J},n_\text {T},n_\text {JT},n_\text {TJ})\rightarrow (n_\text {O},n_\text {J}+1,n_\text {T},n_\text {JT},n_\text {TJ})]\\&\hspace{2cm} = \frac{n_\text {O}b_\text {O}m_{\text {O}\rightarrow \text {J}}}{n_\text {O}b_\text {O}+n_\text {J}b_\text {J}+n_\text {T}b_\text {T}+n_\text {JT}b_\text {JT}+n_\text {TJ}b_\text {TJ}}\\&\hspace{2.6cm} +\frac{n_\text {J}b_\text {J}(1-m_{\text {J}\rightarrow \text {JT}})}{n_\text {O}b_\text {O}+n_\text {J}b_\text {J}+n_\text {T}b_\text {T}+n_\text {JT}b_\text {JT}+n_\text {TJ}b_\text {TJ}}, \end{aligned} \end{aligned}$$

6d $$\begin{aligned}{} & {} \begin{aligned}&{\mathbb {P}}[(n_\text {O},n_\text {J},n_\text {T},n_\text {JT},n_\text {TJ})\rightarrow (n_\text {O},n_\text {J},n_\text {T}+1,n_\text {JT},n_\text {TJ})]\\&\hspace{2cm} = \frac{n_\text {O}b_\text {O}m_{\text {O}\rightarrow \text {T}}}{n_\text {O}b_\text {O}+n_\text {J}b_\text {J}+n_\text {T}b_\text {T}+n_\text {JT}b_\text {JT}+n_\text {TJ}b_\text {TJ}}\\&\hspace{2.6cm} +\frac{n_\text {T}b_\text {T}(1-m_{\text {T}\rightarrow \text {TJ}})}{n_\text {O}b_\text {O}+n_\text {J}b_\text {J}+n_\text {T}b_\text {T}+n_\text {JT}b_\text {JT}+n_\text {TJ}b_\text {TJ}}, \end{aligned} \end{aligned}$$

6e $$\begin{aligned}{} & {} \begin{aligned}&{\mathbb {P}}[(n_\text {O},n_\text {J},n_\text {T},n_\text {JT},n_\text {TJ})\rightarrow (n_\text {O},n_\text {J},n_\text {T},n_\text {JT}+1,n_\text {TJ})]\\&\hspace{2cm} = \frac{n_\text {J}b_\text {J}m_{\text {J}\rightarrow \text {JT}}}{n_\text {O}b_\text {O}+n_\text {J}b_\text {J}+n_\text {T}b_\text {T}+n_\text {JT}b_\text {JT}+n_\text {TJ}b_\text {TJ}}\\&\hspace{2.6cm} +\frac{n_\text {JT}b_\text {JT}}{n_\text {O}b_\text {O}+n_\text {J}b_\text {J}+n_\text {T}b_\text {T}+n_\text {JT}b_\text {JT}+n_\text {TJ}b_\text {TJ}}, \end{aligned} \end{aligned}$$

and

6f $$\begin{aligned} \begin{aligned}&{\mathbb {P}}[(n_\text {O},n_\text {J},n_\text {T},n_\text {JT},n_\text {TJ})\rightarrow (n_\text {O},n_\text {J},n_\text {T},n_\text {JT},n_\text {TJ}+1)]\\&\hspace{2cm} = \frac{n_\text {T}b_\text {T}m_{\text {T}\rightarrow \text {TJ}}}{n_\text {O}b_\text {O}+n_\text {J}b_\text {J}+n_\text {T}b_\text {T}+n_\text {JT}b_\text {JT}+n_\text {TJ}b_\text {TJ}}\\&\hspace{2.6cm} +\frac{n_\text {TJ}b_\text {TJ}}{n_\text {O}b_\text {O}+n_\text {J}b_\text {J}+n_\text {T}b_\text {T}+n_\text {JT}b_\text {JT}+n_\text {TJ}b_\text {TJ}}. \end{aligned} \end{aligned}$$

Note that there are two processes that increase the number of cells with at least one mutation: replication of the cell and a less-mutated mother cell producing a daughter that acquires the necessary mutation.

The three mechanistic regimes we will explore can be described as $b_\text {T}>b_\text {J}$ for Mechanism **(A)**, $m_{\text {O}\rightarrow \text {T}}=m_{\text {J}\rightarrow \text {JT}}>m_{\text {T}\rightarrow \text {TJ}}=m_{\text {O}\rightarrow \text {J}}$ for Mechanism **(B)**, and $m_{\text {J}\rightarrow \text {JT}}>m_{\text {O}\rightarrow \text {T}}$ for Mechanism **(C)**. Examples of birth rate coefficients $(b_\text {O}, b_\text {J}, b_\text {T}, b_\text {JT}, b_\text {TJ})$ and mutation probabilities $(m_{\text {O}\rightarrow \text {J}}, m_{\text {O}\rightarrow \text {T}}, m_{\text {J}\rightarrow \text {JT}}, m_{\text {T}\rightarrow \text {TJ}})$ associated with scenarios **(A)**, **(B)**, and **(C)** are given in Table 2. Here, and in our subsequent analyses and without loss of generality, we assume a common value for all death coefficients $d_\text {O}=d_\text {J}=d_\text {T}=d_\text {JT}=d_\text {TJ}=1$.

**Table 2:** A generalized Moran process is simulated to explore the three different mechanistic scenarios, **(A)**, **(B)**, and **(C)**, with corresponding relative birth coefficients *b* and mutation probabilities *m* listed

	$b_\text {O}$	$b_\text {J}$	$b_\text {T}$	$b_\text {JT}$	$b_\text {TJ}$	$m_{\text {O}\rightarrow \text {J}}$	$m_{\text {O}\rightarrow \text {T}}$	$m_{\text {J}\rightarrow \text {JT}}$	$m_{\text {T}\rightarrow \text {TJ}}$
Mechanism **(A)**	1	2	4	4	4	0.1	0.1	0.1	0.1
Mechanism **(B)**	1	2	2	2	2	0.1	0.2	0.2	0.1
Mechanism **(C)**	1	2	2	2	2	0.1	0.1	0.2	0.1

Death coefficients are equal and set to $d_{i} = 1$. The mutation probability is the probability that one daughter acquires a mutation at birth

These weights and probabilities will be used in our Moran model to describe the evolution of cellular mutation-state subpopulations. Conceptually, we use this Moran process to study observations (**5’, 6’**) at the time that the first double-mutation cell appears, the process is stopped once $n_\text {JT}=1$ or $n_\text {TJ}=1$. At this point, if both $n_\text {J}>0$ and $n_\text {T}>0$, the order of mutation cannot be inferred and the trajectory is not counted. If $n_\text {JT}=1$ and $n_\text {T}=0$, we record the corresponding $n_\text {J}$ and the current time point *T*. This mechanism reflects a JAK2-first patient. If $n_\text {TJ}=1$ and $n_\text {J}=0$, we record the corresponding $n_\text {T}$ and the current time point *T*. This mechanism reflects a TET2-first patient.

### Numerical Computation

The Moran model can be easily simulated via Monte Carlo methods using the exact transition probabilities Eq. 6(a-f). However, if we assume a not-too-large population, we can also use Eqs. 6(a-f) to compute the probabilities of each configuration of the system after each replacement event. For all three scenarios listed in Table 2, we assume an initial population $n_\text {O}=100,n_\text {J}=n_\text {T}=n_\text {JT}=n_\text {TJ}=0$ so that the total population is $n= n_\text {O}+n_\text {J}+n_\text {T}=100$ up until the round at which the first double-mutation cell arises, $n_\text {JT}=1$ or $n_\text {TJ}=1$. For our $n=100$ system, we need to update probabilities over only $(1+100)\times 100/2 = 5050$ configurations (defined by the numbers of JAK2-only or TET2-only cells). We stop the probability updating provided the probability *S*(*t*) that no double-mutation cell has appeared up to time step *t* reaches $S(t) < 10^{-8}$ and $t > 400$. From this conditional survival probability, we find the conditional times to the first appearance of both mutations and the associated number of cells. The expected number of single-mutation cells thus has absolute error no larger than $n S(t) \approx 10^{-6}$, where $n=100$ is the total cell number. By excluding all ending configurations that contain JAK2-only *and* TET2-only cells, we compute the statistics at the double-mutation end-state.

From our computed state probabilities, we can construct relevant quantities such as expectation values and variances. For example, to address observation (**5’**), we can construct and compare ${\mathbb {E}}[n_\text {J}\!\mid \! n_\text {JT}=1,n_\text {T}=0]$ and ${\mathbb {E}}[n_\text {T}\!\mid \! n_\text {TJ}=1,n_\text {J}=0]$, while to investigate observation (**6’**), we compute ${\mathbb {E}}[T\!\mid \! n_\text {JT}=1,n_\text {T}=0]$ and ${\mathbb {E}}[T\!\mid \! n_\text {TJ}=1,n_\text {J}=0]$ and compare their values. Table 3 shows that the expected values of cell populations and mean stopping times are different for JAK2-first cell populations and TET2-first populations. The associated conditional quantiles are listed as $[\ldots ]$ and their expectations and standard deviations (in parentheses). We see that all three mechanistic scenarios can reproduce observations (**5’, 6’**). However, biologically, it is natural to assume that JAK2 and TET2 have appreciably different mutation rates (Mechanism **(B)**). Since mechanisms **(A)** and **(C)** require more supporting evidence, we propose that the scenario associated with **(B)** is sufficient to explain the observations.

**Table 3:** Numerical solution of our Moran process with fixed $n=100$ cells

	(JAK2-first) $[n_\text {J}\!\mid \! n_\text {JT}=1, n_\text {T}=0]$	(TET2-first) $[n_\text {T}\!\mid \! n_\text {TJ}=1,n_\text {J}=0]$	(JAK2-first) $[T\!\mid \! n_\text {JT}=1,n_\text {T}=0]$	(TET2-first) $[T\!\mid \! n_\text {TJ}=1,n_\text {J}=0]$
Mechanism **(A)**	4.03 (3.23)	5.41 (5.00)	24.21 (17.03)	26.05 (18.47)
Mechanism **(B)**	2.34 (1.65)	6.49 (5.09)	11.46 (7.82)	24.68 (17.80)
Mechanism **(C)**	3.59 (2.68)	4.26 (3.53)	22.79 (15.76)	25.79 (18.87)

Expected values and standard deviations (in parentheses) of the one-mutation cell number and the times *T* when the first double-mutation cell appears. For the three different scenarios, **(A)**, **(B)**, and **(C)**, defined in Table 2, we present the corresponding expected values for JAK2-first samples and TET2-first samples

Our results were derived for a system size of $n=100$ cells. Table 3 shows that for all three scenarios, differences in the expected numbers and the mean times to double mutation are consistent with observations (**5’, 6’**). However, the standard deviations indicate appreciable variability and overlap between JAK2-first and TET2-first distributions. Therefore, an appreciable number of observations are required to resolve the differences in numbers and first passage times. For the variance of these quantities in this specific calculation (system size $n=100$), we can estimate the number of samples in each scenario (mechanism **(A)**, **(B)**, or **(C)** and JAK2-first or TET2-first patient) that would pass a *t*-test for detecting a difference in cell numbers or times *T*. For p=0.01, we find that Mechanisms **(A)** and **(C)** require a few hundred samples, while Mechanism **(B)** can be resolved with about dozen samples.

Interestingly, although these results depend on the fixed total population *n* (for which we used $n=100$), we find that the mean and variances are relatively insensitive to system size *n*, with mean numbers $n_\text {J}, n_\text {T}$ and times *T* both modestly decrease with increasing system size *n*, provided $n \gtrsim 40-50$. The expected values asymptote to values somewhat lower than those given in Table 3 as $n \rightarrow \infty$, but they all retain the same *relative* order, explaining observations (**5’, 6’**).

## Discussion and Conclusions

In this paper, we consider two genetic mutations in MPN: JAK2 and TET2. The effect of one mutation depends on whether the other mutation is present. When both the mutations are present, the order of their appearance also affects gene expression. For MPN, the order of the JAK2 V617F and DNMT3A mutations can also affect cellular proliferation (Nangalia et al. 2015). The TET2 and DNMT3A mutations confer epigenetic changes in transcription that are passed on to daughter cells, thus providing a mechanism of “memory” required for bi/multistability and ultimately an order-of-mutation effect. Dependence of cell populations on the order of mutation also appear in other types of cancer. For example, in adrenocortical carcinomas, if the Ras mutation appears before the p53 mutation, the tumor will be malignant and metastatic, but if the p53 mutation appears before the Ras mutation, the tumor will be benign (Herbet et al. 2012). Similar observations can be found in other contexts (Levine et al. 2019; Turajlic et al. 2018; Caravagna et al. 2018).

We constructed several sub-models to explain the mutational patterns and features observed in MPN, specifically addressing observations recorded to date for the JAK2/TET2 mutation pair. In Sect. 3.3, we describe experimental evidence that partially verifies our model. Our models also naturally give rise to predictions that can be tested experimentally. For example, if PRMT5 or E2F1 is knocked out or knocked down, a subsequent JAK2 mutation is predicted to decrease the expression of AURKB, MCM2, MCM4, MCM5, and TK1. Moreover, the expression levels of E2F1 and PRMT5 in cells with a TET2 mutation is predicted to be lower than in cells without the TET2 mutation. Table 4 below summarizes our analysis in terms of the observations it addresses and relative to previous studies. A more detailed outline of these previous investigations is given in Appendix A.

**Table 4:** A summary of studies in mutational order and how they address observations given in Ortmann et al. (2015)

	Key assumptions	Quantitative?	Observation(s) explained
*Previous studies*			
Kent and Green (2017)	JAK2 and TET2 mutations compete for the same regulation region	✗	**(4)**
Kent and Green (2017)	JAK2 and TET2 have different effects on microenvironments	✗	**(4)**
Roquet et al. (2016)	Mutations work like recombinases	✗	**(4)**
Clarke et al. (2019), Talarmain et al. (2022), Talarmain (2021), Mazaya et al. (2020)	Gene expression satisfies a generalized boolean network model with multistability	✓	**(4)**
Ortmann et al. (2015), Ascolani and Liò (2019), Clarke et al. (2019)	JAK2 and TET2 mutations confer different advantages to cell proliferation	✓	**(5)**
Teimouri and Kolomeisky (2021)	JAK2 and TET2 mutations bring different advantages to cell proliferation	✓	**(6)**
*Current analysis*			
ODE model	Gene expression satisfies a nonlinear ODE with bistability	✓	**(1, 2, 3, 4)**
Markov chain model	Gene expression satisfies a Markov chain with bistability	✓	**(4)**
Moran process, Mechanism **(A)**	JAK2 and TET2 mutations bring different advantages to cell proliferation	✓	**(5, 6)**
Moran process, Mechanism **(B)**	Mutation rates for JAK2 and TET2 are different	✓	**(5, 6)**
Moran process, Mechanism **(C)**	JAK2 mutation can induce TET2 mutation	✓	**(5, 6)**

Previous studies (top) and corresponding explanations are compared with the understanding afforded by our proposed mechanisms and models (bottom)

Although we have developed a mathematical framework consistent with all observations to date, there are other possible processes that can lead to the rich set of observations discussed. Potential interactions with the adaptive immune system may inhibit cancer progression (Mellman et al. 2011; Altrock et al. 2015). Cancer may also inhibit the proliferation of white blood cells (Hamanishi et al. 2007), which can lead to multistability in mathematical models of immune response to cancer (Garcia et al. 2020; Li and Levine 2017; Vithanage et al. 2021). Since certain mutations can help cancer cells escape the immune system (Hanahan and Weinberg 2011), it is possible that the order of mutations affects cancer cell populations indirectly by interfering with the immune system. Finally, cancer cells can also affect and be affected by their microenvironments and other cells (through e.g., epigenetically driven “microenvironment feedback”). These nonlinear interactions have been modeled can lead to nonlinear dynamics in relative populations of different cancer cell types (different epigenetic or mutational states) (Smart et al. 2021). Further developing models that incorporate immune and indirect cell-cell interactions could potentially lead to non-additivity and non-commutivity of mutation order in both gene expression and cell population dynamics. Formulating such mathematical frameworks, especially those coupling intracellular state dynamics to epigenetic memory in proliferating cell populations will be the subject of future investigation.

## Figures and Tables

**Fig. 6:**
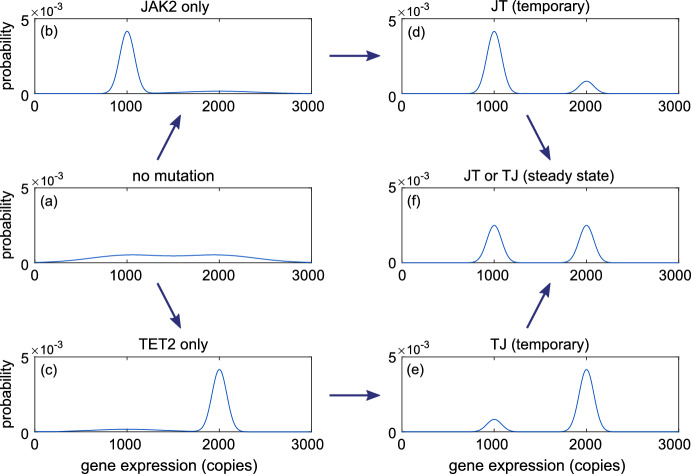
The probability distribution over gene expression levels *x* under the different enumerated scenarios described by the Markov chain model in Appendix B. **a** The stationary distribution when no mutation is present. **b** The distribution of *x* in cells with only a JAK2 mutation. **c** The stationary distribution when only TET2 mutation is present. **d** The temporary (but long-lived) distribution when a TET2 mutation appears on a lineage with an existing JAK2 mutation. **e** Long-lived probability distribution of *X* if the JAK2 mutation appears on a background of existing TET2 mutations. **f** The final distribution when both JAK2 and TET2 mutations are present (regardless of order)

**Fig. 7:**
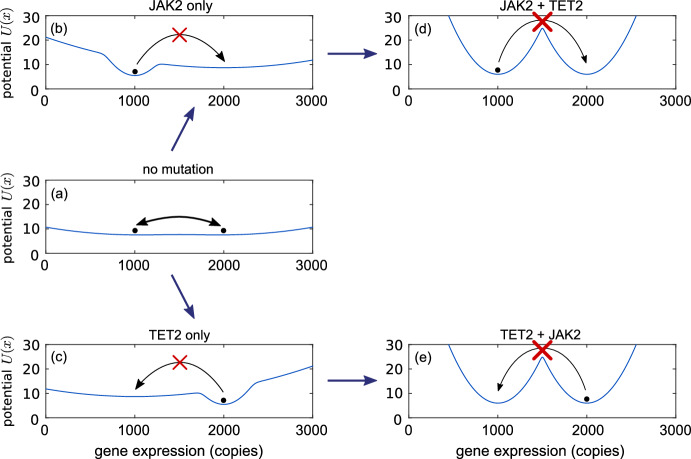
The effective potential function *U*(*x*) of gene expression levels *x* for different scenarios within the Markov chain model. **a** The potential function in the absence of mutations. The system can switch between the shallow wells near $x=1000$ and $x=2000$. **b** *U*(*x*) when only the JAK2 mutation is present. The system is confined to the deep well near $x=1000$. **c** *U*(*x*) when only the TET2 mutation is present. The system is confined to the deep well near the higher expression level $x=2000$. **d** The potential function of X gene expression if the TET2 mutation appeared after the JAK2 mutation. Since the system was previously confined in the well near $x=1000$, it will retain this lower level of expression for a long time, potentially over a typical lifetime. **e** *U*(*x*) for a cell which acquired the JAK2 mutation from a mother cell which already had the TET2 mutation. Since the system was previously confined in the well near $x=2000$, its gene expression will stay high for a long time. Theoretically, in the (unrealistically) long time limit, the probability densities in both cases **d** and **e** will equipartition itself symmetrically across the two equally deep wells

## Previous Models and Comparisons

Here, we summarize the previous models for such clinical observations (Ortmann et al. 2015; Swanton 2015; Roquet et al. 2016; Kent and Green 2017; Ascolani and Liò 2019; Clarke et al. 2019; Talarmain et al. 2022; Talarmain 2021; Mazaya et al. 2020; Teimouri and Kolomeisky 2021) and compare them with our proposed conceptual framework.

Ortmann et al. (2015) assume that TET2 mutation can significantly increase the proliferation rate of cancer stem cells, while JAK2 mutation only has a weak growth advantage. Therefore, for TET2-first patients, TET2-only cells first spread, and TET2-JAK2 cells (which do not have a significant growth advantage over TET2-only cells) do not dominate. For JAK2-first patients, JAK2-only cells do not spread that much, while JAK2-TET2 cells (after they appear) can dominate. Therefore, TET2-first patients have a much higher percentage of cells with only one mutation, consistent with observation **(5)**. In Sect. 4, we also discuss that these assumptions are consistent with observation **(6)**. See also the interpretation by Swanton (2015).

Kent and Green (2017) propose two explanations for observation **(4)**.

(i) Both JAK2 and TET2 mutations can participate in epigenetic regulation (Dawson et al. 2009; Ito et al. 2010; Shih et al. 2012), but the regulation mechanism might be incompatible. For example, the first mutation might lead to the occlusion of certain genomic regions, so that the second mutation cannot regulate genes in those regions. This mechanism would lead to $x^*_\text {JT}= x^*_\text {J}\ne x^*_\text {T}= x^*_\text {TJ}$, which is not consistent with other observations.

(ii) For either JAK2-first or TET2-first patients, before the appearance of the second mutation, different first mutations might lead to different cell types and abundances, leading to different microenvironments in which double mutant cells that subsequently arise find themselves. This indirect effect can also shape disease progression.

Although these concepts explain the non-commutative order-of-mutation effects (observation **(4)**), they invoke hypothetical mechanisms that lack experimental evidence.

In a related study, Roquet et al. (2016) consider the effect of recombinases (i.e., genetic recombination enzymes) on gene sequences. When applying different recombinases to gene sequences, their order of application can lead to different results. For example, consider a gene sequence 12312 and two recombinases A, and B. Suppose A deletes genes between the “1s”, and B inverts genes between the two “2s” (if there are not two “2s”, B does nothing). If the DNA is exposed to A before B, the gene sequence becomes

$$\begin{aligned} 12312\xrightarrow []{\texttt {A}}112\xrightarrow []{\texttt {B}}112. \end{aligned}$$

If A is added after B, the gene sequence becomes

$$\begin{aligned} 12312\xrightarrow []{\texttt {B}}12132\xrightarrow []{\texttt {A}}1132. \end{aligned}$$

Although this system describes rearrangements and not specific mutations, it nonetheless provides a possible mechanism for observation **(4)**. These mathematical operators are not commutative but are not connected to genetic mutations highlighted in cancer progression.

Ascolani and Liò (2019) constructed a complex cellular automata model to describe cancer metastasis. They assume that different mutations lead to different proliferation and/or apoptosis rates. This assumption, as was invoked by Ortmann et al., can explain observation **(5)**. However, although Ascolani and Liò also take into account how different *orders* of different mutations can affect proliferation and apoptosis differently, they did not explicitly apply their model to the observations associated with different orders of JAK2 and TET2 mutations in the MPN system.

Clarke et al. (2019) model the gene regulatory network as a generalized boolean network that evolves under certain rules and exhibits fixed points and/or limit cycles for gene expression levels. Each mutation fully activates or inhibits one gene (the expression level is fixed, similar to the do-operator used in causal inference (Benferhat and Smaoui 2007)), thus changing the fixed points and/or limit cycles. Certain combinations of mutations lead to higher proliferation rates or apoptosis rates, making these mutation patterns more likely or less likely, respectively. There might be multiple fixed points and/or limit cycles, and different orders of mutations might lead to different final states of gene expression. Different sequences of perturbations leading to different states have been hypothesized for different physiological dynamics, including neuroendocrine stress response (Kim et al. 2018; Cheng et al. 2021). This type of model can be used to explain observations **(4)** and **(5)**.

Talarmain et al. (2022); Talarmain (2021) apply the model in Clarke et al. (2019) paper to the JAK2/TET2 mutation order problem to explain observation **(4)**. They find a concrete generalized boolean network of gene expression. Mutations can affect the dynamics of this network. When there is no mutation or just one mutation, the system has one stable fixed point. When both JAK2 and TET2 mutations are present, the system has two stable fixed points. Different orders of mutations lead to different fixed points. The Talarmain et al. model invokes a third, downstream gene HOXA9 which is directly affected by different orders of JAK2 and TET2 mutations, which then affects many other downstream genes.

Mazaya et al. (2020) use a Boolean network to model the effect of mutations. The model dynamics and the explanation of observation **(4)** are similar to that of Clarke et al.’s model. Mazaya et al. further analyze this model to study when the network is more sensitive to different orders of mutations.

Clarke et al. (2019), Talarmain et al. (2022); Talarmain (2021), and Mazaya et al. (2020) all use (generalized) Boolean networks with a discrete state space and artificially chosen parameters to explain observation **(4)**.

Finally, Teimouri and Kolomeisky (2021) use a random walk model to study the acquisition of two mutations. The first stage is a random walk on $0,1,\ldots ,n$, representing the number of cells with the first mutation. The first stage starts at 0, and finishes when reaching *n*, meaning that all *n* cells have the first mutation. The process terminates if reaching 0 again before reaching *n*. The second stage is a random walk on $n,n+1,\ldots ,2n$, representing the number of cells with the second mutation plus *n*. The second stage starts at *n*, and finishes when reaching 2*n*, meaning that all cells have both mutations. If the process reaches 2*n*, they count the total time and take the expectation. Teimouri and Kolomeisky (2021) prove that if the first mutation has a higher fitness than the second mutation, then the tumor formation probability is higher, but the time for tumor formation is longer. This framework can directly explain observation **(6)** but the second mutation can appear if and only if the first mutation rapidly expands in the cell population. Because they assume that for all cells arriving at the final two-mutation state is conditioned on no extinction once a mutation appears, they underestimate the predicted time to reach the final state.

Our suite of mathematical models are compared to these previously proposed concepts in Table 4. Our models provide better coverage of the observed phenomena and can be concatenated for a more complete picture of MPN progression. We now provide an overview of our more complete analysis, filling in some mechanistic explanations of observations **(1–6)**.

Observations **(1)** and **(2)**, the up-regulation of certain genes depending on the presence and absence of certain mutations, form a common “logic gate” in which expression levels can be changed if and only if both conditions are met. Our simple nonlinear ODE models not only explain observations **(1, 2)** but are also building blocks for a model to explain observation **(3)**, for which no model has thus far been proposed. In explaining **(3)**, we find two candidates for a hidden factor. Some regulatory terms in our model have been verified experimentally, and we propose experiments to examine other regulatory relationships. Finally, our nonlinear, bistable ODE models provide an inherently natural framework for non-commutative order-of-mutation effects on gene expression (observation **(4)**), while conforming to all other observations and physiological postulates.

To study observations **(5, 6)**, we consider a generalized Moran process, which is a more realistic model for describing the population dynamics of hematopoietic stem and progenitor cells. We find that three different parameter limits, describing three distinct biological mechanisms (including that proposed by Teimouri and Kolomeisky) can reproduce observations **(5, 6)** separately.

## Markov Chain Model for Observation **(4)**

In the nonlinear ODE models developed for observation **(4)** (for example, $\lambda = 2+\mathbb {1}_{\textrm{J}}-\mathbb {1}_{\textrm{T}}$), a cell with no mutation and a cell with both mutations reside on the same gene expression landscape. Since gene expression at the single-cell level is essentially stochastic, we can also build an *ad hoc* Markov chain model in which the landscape of gene expression changes with the appearance of each mutation.

In a single cell, the expression level (protein or mRNA count) of gene X is a nonnegative integer random variable *X*. Here, we shall assume *X* is sufficiently abundant that we can approximate all distributions and rates by functions continuous in *x* but that can be defined only at integer values. We first define the distribution of rates

7 $$\begin{aligned} \begin{aligned} A_1(x,c_1,\mu _1,\sigma _1)=&\frac{c_1}{\sqrt{2\pi \sigma _1^2}}e^{-\frac{(x-\mu _1+1)^2}{2\sigma _1^2}}\!\!\!\!, \quad \, B_1(x,c_2,\mu _2,\sigma _2) =\frac{c_2}{\sqrt{2\pi \sigma _2^2}}e^{-\frac{(x-\mu _2+1)^2}{2\sigma _2^2}},\\ A_2(x,c_1,\mu _1,\sigma _1) =&\frac{c_1}{\sqrt{2\pi \sigma _1^2}} e^{-\frac{(x-\mu _1)^2}{2\sigma _1^2}}\!\!\!\!, \qquad B_2(x,c_2,\mu _2,\sigma _2) = \frac{c_2}{\sqrt{2\pi \sigma _2^2}}e^{-\frac{(x-\mu _2)^2}{2\sigma _2^2}}. \end{aligned} \end{aligned}$$

*X* follows a continuous-time Markov chain on $0,1,2,\ldots$ where the transition rate from *x* to $x+1$ is defined as $r_{x\rightarrow x+1}=A_1(x)+B_1(x)$, and the transition rate from $x+1$ to *x* is defined as $r_{x+1\rightarrow x}=A_2(x)+B_2(x)$. Since this Markov chain has no cycles, a detailed balance condition is satisfied Wang and Qian (2020) and we can directly compute the equilibrium probability distribution ${\mathbb {P}}(X=x)$ from the detailed balance condition

8 $$\begin{aligned} {\mathbb {P}}(X=x)r_{x\rightarrow x+1}={\mathbb {P}}(X=x+1)r_{x+1\rightarrow x}. \end{aligned}$$

The $\mu _{1}, \mu _{2}$ parameters determine the peaks of the rates while $c_{1}, c_{2}$ determine the relative magnitude of the peaks of the rates. The equilibrium probabilities ${\mathbb {P}}(X=x)$ from Eq. 8 shows that the probability accumulates around mean values $\mu _{1}$ and $\mu _{2}$, while the ratios of the heights at each peak are defined by $c_{1}/c_{2}$.

In all cases, we set $\mu _1=1000$, $\mu _2=2000$. If the JAK2 mutation is not present, we set $c_1=1/\tau$ and $\sigma _1=400/\tau$ in $A_1(x,c_1,\mu _1,\sigma _1)$ and $A_2(x,c_1,\mu _1,\sigma _1)$; otherwise, when the JAK2 mutation is present, we set $c_1=5/\tau$ and $\sigma _1=80$. Similarly, if TET2 mutation is not present, we set $c_2=1/\tau$ and $\sigma _2=400$ in $B_1(x,c_2,\mu _2,\sigma _2)$ and $B_2(x,c_2,\mu _2,\sigma _2)$; otherwise, when the TET2 mutation is present, we set $c_2=5/\tau$ and $\sigma _2=80$. In this scenario, a JAK2 mutation makes the mRNA count more concentrated near $x=1000$ (The peak near 1000 is higher and sharper), while a TET2 mutation sharpens the mRNA count around $x=2000$. When both mutations arise, the two peaks have the same height but are both sharper than the peaks of wild-type expression. While the timescales for a mutation to occur are not modeled in this process, after mutation, the relaxation of intracellular gene dynamics, to their new steady states occur with rates $c_{1}$ and $c_{2}$ defined in terms of the typical intracellular gene dynamics timescale $\tau$, on the order of seconds to minutes.

The probabilities are plotted in Fig. 6. If no mutation is present, the stationary distribution is rather flat with two low peaks near $x=1000$ and $x=2000$, as shown in (a). If only the JAK2 mutation is present, the stationary distribution is mostly concentrated in a sharp peak near $x=1000$, with a small flat probability peak near $x=2000$ develops after $\sim 10^5 \tau \sim \textrm{month}$, as depicted in (b). If the TET2 mutation appears after the JAK2 mutation, the small flat probability peak near $x=2000$ first sharpens to a more localized peak near $x=2000$ (d); only after an extremely long time (e.g., $10^8 \tau \sim$ lifetime), the heights of two peaks near $x=1000$ and $x=2000$ equalize, as shown in (f). If only the TET2 mutation is present, the stationary distribution shown in (c) is mostly concentrated in a sharp peak near $x=2000$, with a small nearly flat probability mound near $x=1000$. If the JAK2 mutation then appears, the small broad probability peak near $x=1000$ first shrinks on a fast timescale ($\sim 10^5 \tau \sim \textrm{month}$) to a sharper peak near $x=1000$ (e); only after an extremely long time, the heights of two peaks near $x=1000$ and $x=2000$ again equilibrate to the common double-mutation steady state shown in (f). Thus, when considering finite times, this model yields an apparent order-of-mutation affect ((d) *vs.* (e)).

We can also define a potential at $X=x$ as the negative logarithm of the stationary distribution: $U(x)=-\log {\mathbb {P}}(X=x)$. Figure 7 shows the potential function *U*(*x*) corresponding to different temporal configurations of mutations. If no mutation is present, the potential has two shallow wells near $x=1000$ and $x=2000$. The expression level can easily move between these two wells, as shown in Fig. 7a. Figure 7b depicts the case in which only the JAK2 mutation is present for which there is a deep well near $x=1000$ and a shallow well near $x=2000$. Here, it is easy to jump from the shallow well into the deep well, but not the other way around. Thus, this system is most likely to have expression level $x=1000$. If the TET2 mutation appeared after the JAK2 mutation (Fig. 7d), the system will first stay in the deep well near $x=1000$. Since both wells are deep, there is very little probability flux from one well to another and the probability distribution relaxes very slowly (potentially longer than a lifespan) towards final equipartition. If only the TET2 mutation is present, the system is likely to stay in the deep well near $x=2000$, indicated in Fig. 7c. Finally, if the JAK2 mutations appear after TET2 mutations, the system will reside in the well near $x=2000$ (see Fig. 7e) before very slowly becoming equally distributed between $x\approx 1000$ and $x\approx 2000$.

In this model, different orders of mutations (JT and TJ) lead to the same final stationary distribution (Fig. 6f). However, different histories lead to concentration of probabilities to different wells (Fig. 6d, e) on finite timescales. If this mesoscopic timescale is comparable to the life span of human being, then the final stationary distribution is *de facto* inaccessible. This simple probabilistic model can also explain the difference in gene expression levels between patients with different orders of mutations.
